# Assessing the impact of isolation policies on epidemic dynamics through swarm entropy

**DOI:** 10.3389/fpubh.2024.1338052

**Published:** 2024-02-08

**Authors:** Junxiao Xue, Yihang Guo, Mingchuang Zhang

**Affiliations:** ^1^School of Cyber Science and Engineering, Zhengzhou University, Zhengzhou, China; ^2^Zhejiang Lab, Research Institute of Artificial Intelligence, Hangzhou, China; ^3^College of Computer Science and Technology (CCST), Zhejiang University (ZJU), Hangzhou, China; ^4^School of Intelligent Science and Technology, Hangzhou Institute for Advanced Study of University of Chinese Academy of Sciences (UCAS), Hangzhou, China; ^5^National Digital Switching System Engineering and Technological R&D Center, People's Liberation Army Strategic Support Force Information Engineering University, Zhengzhou, China

**Keywords:** pandemic forecasting, swarm entropy, SIR model, policy, infectious diseases

## Abstract

Isolation policies are an effective measure in epidemiological models for the prediction and prevention of infectious diseases. In this paper, we use a multi-agent modeling approach to construct an infectious disease model that considers the influence of isolation policies. The model analyzes the impact of isolation policies on various stages of epidemic from two perspectives: the external environment and agents behavior. It utilizes multiple variables to simulate the extent to which isolation policies influence the spread of the pandemic. Empirical evidence indicates that the progression of the epidemic is primarily driven by factors such as public willingness and regulatory intensity. The improved model, in comparison to traditional infectious disease models, offers greater flexibility and accuracy, addressing the need for frequent modifications in fundamental models within complex environments. Meanwhile, we introduce “swarm entropy” to evaluate infection intensity under various policies. By linking isolation policies with swarm entropy, considering population structure, we quantify the effectiveness of these isolation measures. It provides a novel approach for complex population simulations. These findings have facilitated the enhancement of control strategies and provided decision-makers with guidance in combating the transmission of infectious diseases.

## 1 Introduction

The outbreak and spread of infectious diseases cause significant threats to human society, making effective prevention and control of these diseases a crucial public health concern. To formulate effective prevention and control strategies, it is essential to have a clear understanding of the dynamic changes in infectious diseases. These dynamic changes are influenced by various factors, and one controllable factor is quarantine policies. The purpose of quarantine policies is to reduce contact between infected individuals and susceptible individuals, thereby lowering the risk of transmission. However, these policies also impact people's daily lives and social activities. Therefore, it is worth exploring how to assess the impact of different quarantine policies on the dynamic changes of infectious diseases.

In recent years, group simulation has found widespread applications in various fields, including urban planning, disaster prevention and control, and visual effects (1). By simulating interactions and information exchange among individuals, group simulation can provide data support for formulating relevant management strategies.

The fidelity of group simulation models has been a time-consuming challenge to address. Cunha et al. (2) introduced a data-driven machine learning framework that employs the cross-entropy method to enhance the fidelity of real-time infectious disease models. Kumar and Susan (3) suggests a fuzzy time series (FTS) forecasting method based on particle swarm optimization (PSO) to enhance the accuracy of predictions. The highly realistic data has enhanced the flexibility in choosing modeling methods for simulation experiments, such as agent-based models and system dynamics, this renders the improved infectious disease model more flexible. For example, Contoyiannis et al. (4) introduced a self-organizing mechanism-based infectious disease model capable of adapting to various epidemic transmission scenarios.

Currently, group simulation experiments commonly adopt agent-based modeling, where the behavior of agents is influenced by the external environment and individual factors (5, 6), allowing for individual variations. Researchers have attempted to incorporate knowledge from psychology and dynamics into the behavior models of agents. For instance, in disaster emergency scenarios, emotional contagion has been applied. By defining how panic emotions affect the path planning of agents, a framework for simulation experiments under different situations such as earthquakes and fires can be established eliminating the need for frequent model changes (7).

During the COVID-19 pandemic, various infectious disease models have been proposed to assess important parameters such as infection rates and mortality rates in response to the real situation (8). Das et al. (9) presented an improved infectious disease model that considered heterogeneous populations, including asymptomatic carriers. They compared the effectiveness of two preventive measures: social distancing and isolation. Yang et al. (10) addressed population movement between different regions and utilized artificial agents to analyze the pandemic's overall trend. Zheng et al. (11) further enhanced the accuracy of pandemic forecasting by integrating AI models with infectious disease models. These models have provided clear guidance for epidemic prevention and control efforts and have led to the formulation of a series of preventive measures. To effectively control the pandemic, social distancing and isolation policies should be regarded as primary strategies.

Indeed, implementing isolation policies requiresa consideration of the local healthcare facilities, economic factors, and residents' willingness to comply. For example, a during the pandemic in Shanghai, the isolation policies not only aimed to reduce contact rates with infected individualsa but also placed significant emphasis on residents' mental wellbeing to prevent panic emotions from affecting the efficiency of epidemic prevention and control efforts. In some underdeveloped regions, medical resources may not be sufficient to meet the theoretical requirements for social distancing and isolation. Therefore, when formulating isolation policies, factors like the loss of supplies during distribution should be taken into account. Moreover, different infectious diseases and different stages of the same disease may require the adoption of different isolation policies (12). Relying solely on controlling social distancing might not be sufficient to meet the practical demands in such cases. Thus, a comprehensive and adaptable approach should be employed to address the complexities and variations that arise during epidemic control efforts.

Traditional infectious disease models often evaluate isolation policies by controlling social distancing, which essentially alters the contact rates between agents, affecting the efficiency of information exchange among them. However, based on the theory of group entropy, the process of information interaction is influenced by four aspects: transmission efficiency, population structure, agents behavior, and the external environment. Therefore, equating the control of social distancing with isolation policy efficiency cannot accurately assess complex isolation policies. This paper aims to treat the isolation policy as a comprehensive external environmental variable and analyze the impact of the external environment and individual differences on the transmission process during the epidemic. By establishing an infectious disease transmission model affected by isolation policies, the model can simulate the spread of the epidemic under the influence of isolation policies and evaluate different isolation strategies.

Our main contributions are as follows:

- We proposed a modified infectious disease model, it takes into account the impact of isolation policies on the health status of individuals (agents) and considers the heterogeneity of residents under isolation policies.
- The model provides a method to quantitatively evaluate policy efficiency, avoiding frequent model changes, and is more available for analyzing the development trends of epidemics. This approach can also be applied to other scenarios involving group simulation models.
- The experiments utilized an agent-based modeling approach. The characteristics of agents reflect the diversity and heterogeneity of a coupled network. Additionally, the experiments employed the concept of structure entropy from swarm entropy to systematically analyze the infection efficiency of each stage of the epidemic under different isolation policies, it enables isolation policies to be measured as a parameter.

The remaining sections of the paper are as follows: Section 2 introduces related work on simulation experiments. Section 3 presents the methods used for simulation modeling, with a focus on the improvement of the SIR model and the construction of the environmental model. Section 4 discusses the experimental results and parameters, including control experiments, sensitivity analysis experiments, and simulation validation against real-world situations. The final Section summarizes the main contributions and limitations of this paper.

## 2 Related work

### 2.1 SIR model

In traditional infectious disease models, the population within the scope of disease transmission is divided into three categories: susceptible individuals (S), infected individuals (I), and removed individuals (R) (13). Among these, S represents the group that is susceptible to infection due to lacking immunity, I represents the infectious group capable of transmitting the disease, and R represents the group that has been either cured or deceased and is no longer susceptible to or capable of infecting others. Individual states undergo linear transitions among these three states of susceptible, infected, and removed. The SIR model has been widely applied in infectious disease research. Based on the SIR model, various variant models have been developed according to different social environments and virus characteristics. For example, the SEIR model (14) takes into account the exposed period, and the SIRS model (15) allows individuals to become infected again after recovery. Additionally, the SIR model has found applications in other fields. For instance, Kalimzhanov et al. (16) examined the interaction between the diffusion processes and structural stability in social networks. Mao et al. (17) established an emotional contagion model in group simulations based on the SIR model. Woo et al. (18) simulated the opinion propagation process on the Internet using the SIR model.

### 2.2 Swarm entropy

Swarm entropy is a metric used to measure the heterogeneity or level of disorder within a population, reflecting the uniformity or diversity of individual states within the group. In the analysis of collective agents behavior in biological populations, swarm entropy incorporates the concept of entropy theory into the quantitative analysis of agents behavior in group systems, considering aspects such as information transmission, system structure, and behavioral mechanisms (19). In the context of swarm simulation, swarm entropy equates the behavior of agents to local information interactions, where this information exchange is constrained by external environmental factors and internal behavioral evolutionary dynamics.

Swarm entropy comprises four components: environmental entropy, behavioral entropy, structural entropy, and transmission entropy (20). Environmental entropy accounts for the influence of the physical environment on the agents. Behavioral entropy considers the diversity of agents, such as how individual characteristics impact the efficiency of information transmission. Structural entropy takes into account the influence of the agent group's structure. Transmission entropy, on the other hand, considers the learning capabilities of the agents.

In existing experiments, the entropy increase effect has been used as a means to analyze model efficiency. For instance, Chen (21) applied the concept of swarm entropy to complex software development involving unmanned collectives, Nie et al. (22) discovered a correlation between information entropy and infection rates, which can be applied to the SEIR model.

In this paper, the isolation policies primarily act on the simulation model by altering the way infected and healthy populations interact. Therefore, the efficiency of the infection model can be analyzed using structural entropy. In the simulation experiments, different agents are assigned different identities, and when isolation policies are implemented, the variations in identities lead to changes in the population's structure (23), resulting in different levels of swarm entropy for various population structures. The paper contends that higher swarm entropy indicates greater disorder or diversity in the individual states within the population, implying increased opportunities for contact between infected and susceptible individuals and consequently higher infection intensity. Conversely, lower swarm entropy indicates a more uniform or homogeneous distribution of individual states within the population, resulting in reduced chances of contact between infected and susceptible individuals and thus lower infection intensity.

### 2.3 Regulatory intensity and control rate

Social distancing is a primary measure implemented during pandemics to prevent the spread of infectious diseases. It aims to control the range of people's movement and limit it as much as possible, thereby suppressing the extent and intensity of virus transmission. Its essence lies in influencing the contact rate to reduce the infection rate among susceptible individuals (24). The effectiveness of social distancing depends on the degree and timing of policy implementation, Thada et al. (25) introduced an additional exposure state (E) to distinguish between the isolated state and the regular infectious state.

To ensure variability in experimental results, different isolation policies will be employed for epidemic transmission simulations. However, different isolation policies may lead to changes in population structure. For instance, during the COVID-19 pandemic, updated versions of the SICR model with isolation stages were introduced to adapt to the evolving transmission environment over time. Therefore, this paper assesses the effectiveness of isolation policies by analyzing changes in population structure, using swarm entropy to analyze variations in the entropy of population structure. Additionally, two variables, regulatory intensity, and control rate, are introduced to simulate the impact of external environment and internal factors on the effects of isolation policies on the transmission process.

Regulatory intensity represents the extent to which isolation policies affect the contact rate. Taking into account the population structure, the initial infection rate is set to the infection rate at a social distance of 5 meters, which is determined based on government recommendations for safe distancing during the COVID-19 pandemic. On the other hand, the control rate represents the proportion of individuals constrained by the isolation policy within the population, influenced by asymptomatic carriers and the willingness of agents to comply.

## 3 Methodology

**Figure 1:**
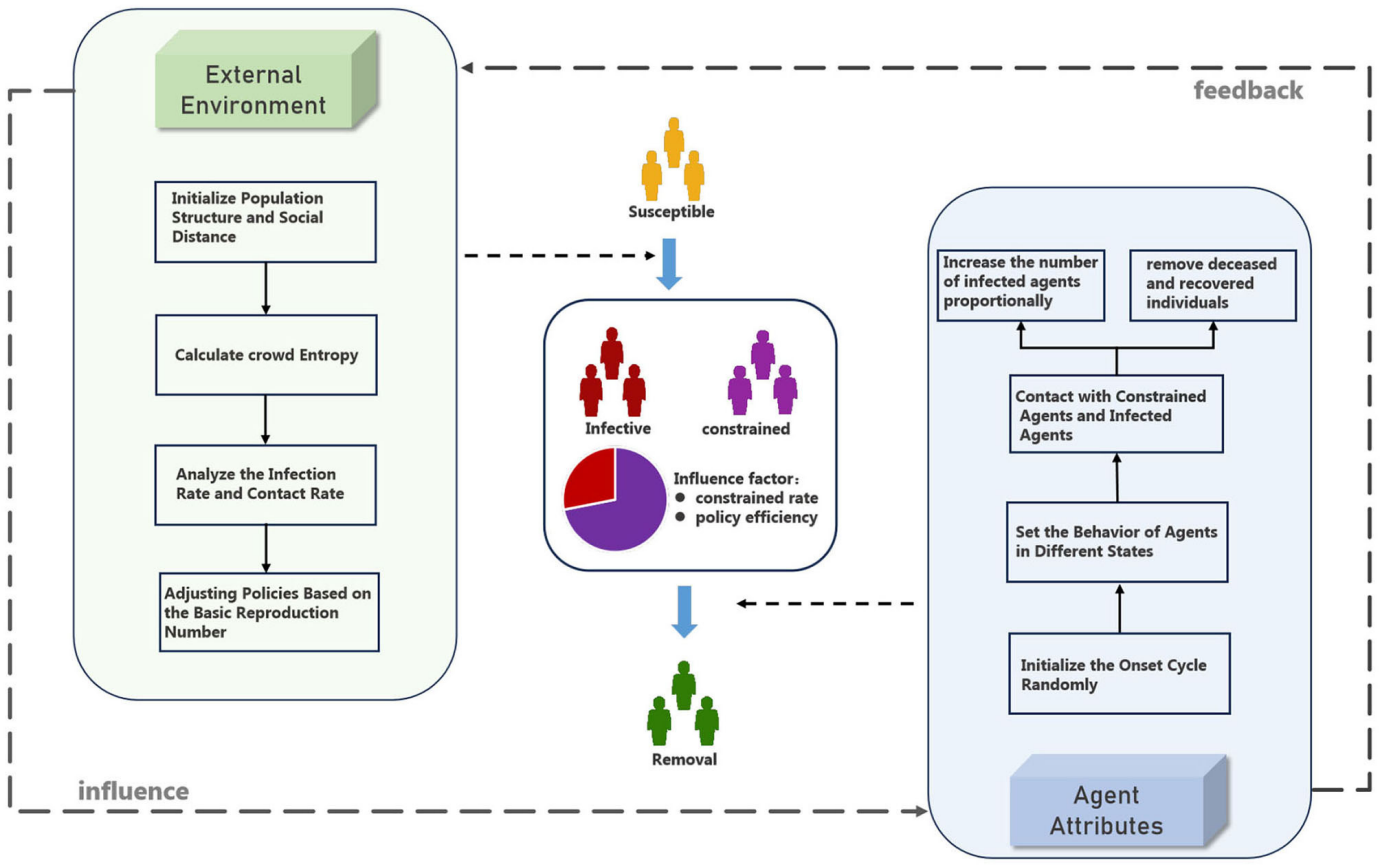
Framework of infection mechanism (in SICR model, we analyze the process of infection from a simulation experiment perspective, enhancing the traditional SIR model by considering both external environmental factors and the attributes of agents).

The epidemic transmission module has established an improved SICR model. In this model, in addition to the conventional susceptible individuals (S), infected individuals (I), and removed individuals (R), a new group of individuals affected by the isolation policy is introduced, referred to as constrained individuals (C). As shown in Figure 1, this group undergoes changes based on the implementation of the isolation policy, including alterations in their infection rate and contact rate. Through the SICR model, the impact of isolation policies on epidemic transmission can be analyzed from both external environmental and agents behavior perspectives (26). External behavior is primarily represented by the impact of isolation policies, which is reflected in the infection stages of the pandemic. Agents behavior is manifested through disease progression and individual preferences, primarily influencing the recovery phase of the pandemic. To evaluate the effectiveness of isolation policies, the simulation module incorporates metrics such as swarm entropy and the basic reproduction number, used to analyze the rationality of the efficiency of isolation policies.

### 3.1 Modified SIR model

The improved SIR model possesses the following three main characteristics:

- Introduction of constrained individuals: in the infectious disease model, a new group of individuals called “constrained individuals” is introduced, who are subject to various degrees of constraint due to isolation policies. Typically, the infectivity and contact rate of constrained individuals is lower than those of regular infected individuals.
- Incorporation of regulatory intensity and control rate: to evaluate the effectiveness of different isolation policies, the experiment introduces the variable “Regulatory intensity” (*Q*_*K*_). It also considers the willingness of asymptomatic carriers and residents, incorporating the “Control Rate” (A). The combined effect of regulatory intensity and control rate determines the impact of isolation policies on the infection process.
- Unified consideration of removed individuals: to simplify the model structure, removed individuals are unified and considered. This group includes individuals who have died, recovered, or lost the ability to infect due to other circumstances.

The traditional SIR virus propagation model reveals the relationships among susceptible individuals, infected individuals, and recovered individuals. This model is a commonly used classic model in epidemiology to describe the transmission process of infectious diseases within a population, encompassing the growth of infected and recovered individuals. As shown in Equations 1, 2.


(1) $$\begin{array}{l} S+I+R=\Omega \end{array}$$

(2) $$\begin{array}{l} \{\begin{array}{c} \frac{△S}{△T}=\beta SI-\frac{△I}{△T},\\ \frac{△I}{△T}=\beta SI-\lambda I,\\ \frac{△R}{△T}=\lambda I. \end{array} \end{array}$$

where the recovery rate of an infected individual is defined as γ, Ω is defined as the total population size. and β is defined in terms of the contagiousness of the disease and the rate of exposure of susceptible individuals to infected individuals, $\sigma =\frac{\beta }{\gamma }$, σ represents the basic reproduction number of a disease. The process of a susceptible individual's movement from illness to emigration can be expressed by a differential equation as Equation 3:


(3) $$\begin{array}{l} I=\left(S_{0}+I_{0}\right)-S+\frac{1}{\sigma }\ln\frac{S}{S_{0}} \end{array}$$

The above differential equation expresses the rate of change of susceptible individuals. The rate of change of susceptible individuals is dependent on the number of infected individuals and the number of susceptible individuals. Specifically, the decrease in the number of susceptible individuals depends on the contact rate between infected and susceptible individuals (β) and the proportion of susceptible individuals. This differential equation describes the dynamics of susceptible individuals over time and is an essential equation in the SIR model for describing the susceptible state.

During the spread of an epidemic, a primary measure taken when the virus begins to proliferate is the isolation of infected individuals to cut off the transmission pathways and control the spread of the virus. However, in practice, when isolation measures are implemented, only individuals with evident symptoms can be identified and isolated promptly. Some agents may lack identifiable infection characteristics, yet these unquarantined individuals can still serve as sources of transmission, these individuals are referred to as asymptomatic carriers. Additionally, there could be some sources of infection that go unnoticed due to other reasons, posing a hidden risk for virus transmission.

The presence of these asymptomatic carriers and undetected sources of infection makes it challenging for isolation policies to achieve comprehensive and effective coverage. Consequently, some individuals may directly transition from the infected state to the removed state without undergoing the isolation process. This highlights why, in real-world scenarios, the spread of an epidemic is influenced not only by isolation policies but also by other factors.

For example, in the later stages of the COVID-19 pandemic, many regions implemented open policies to ensure the normal functioning of society as much as possible. Such low-intensity isolation policies led to the possibility that infected individuals restricted by these policies could still transmit the virus to uninfected individuals. Therefore, different isolation policies can have varying degrees of impact on the transmission process. This highlights the crucial importance of considering various factors when formulating isolation policies. In epidemic prevention and control, understanding the effects and limitations of isolation policies is essential for scientifically devising and implementing more effective control measures.

In the experiments conducted in this paper, only the impact of isolation policies on the infectious disease model is simulated as an external environmental variable. To avoid excessive complexity in the model, the group of individuals constrained by isolation policies is defined as the “constrained individuals”.

In the epidemic simulation, open policies, individual isolation, and community isolation are commonly implemented epidemic prevention measures. Under individual isolation policies, infected individuals can transmit the virus to others through contact with cohabitants. Different policies have distinct effects on the contact rate. In the SICR model, the variables *Q*_*K*_ and A will be used to simulate the effects of different isolation policies.

**Figure 2:**
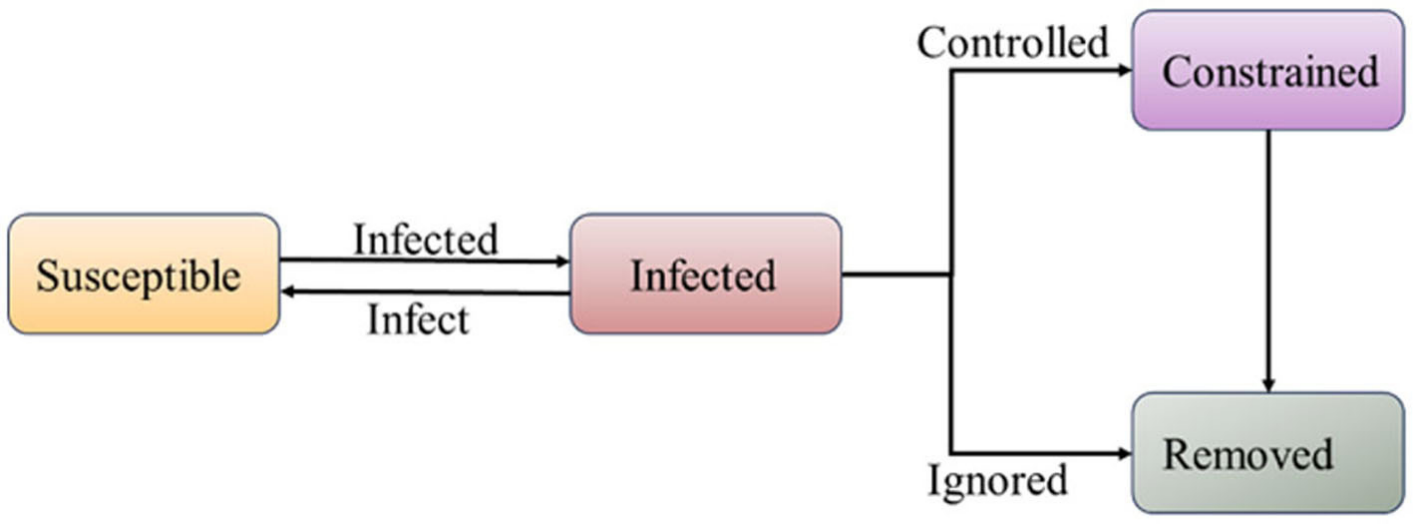
SICR infection model (compared to the SIR model, under the influence of quarantine policies, infected individuals have a certain probability of transitioning into a constrained population).

In the context of epidemic environments with isolation policies in place, the transmission model is divided into four stages: susceptible individuals, infected individuals, constrained individuals, and removed individuals, as shown in Figure 2. Unlike the traditional SIR model, R includes both the recovered group and the infected individuals who are fully isolated and no longer capable of transmitting the virus. Constrained individuals represent individuals who, despite being constrained by isolation policies, can still transmit the virus to others through certain means.

The model setting and stage division enable the simulation to more accurately simulate the effects of different isolation policies on the transmission process, thus providing a useful reference for the development of more effective anti-epidemic measures. The speed of its transformation is expressed by the Equations 4–8:


(4) $$\begin{array}{l} P\left(t\right)=S\left(t\right)+I\left(t\right)+C\left(t\right)+R\left(t\right) \end{array}$$

(5) $$\begin{array}{l} \frac{dS}{dT}=\frac{\left(\sigma \beta I\left(t\right)+\sigma_{1}\beta_{1}C\left(t\right)\right)S\left(t\right)}{P\left(t\right)} \end{array}$$

(6) $$\begin{array}{l} \frac{dI}{dT}=\frac{\left(\sigma \beta I\left(t\right)+\sigma_{1}\beta_{1}C\left(t\right)\right)S\left(t\right)}{P\left(t\right)}-\gamma I\left(t\right)-A\alpha I\left(t\right) \end{array}$$

(7) $$\begin{array}{l} \frac{dC}{dT}=A\alpha I\left(t\right)-\gamma C\left(t\right) \end{array}$$

(8) $$\begin{array}{l} \frac{dC}{dT}=\left(1-A\right)\alpha I\left(t\right)+\gamma C\left(t\right) \end{array}$$

$\frac{dR}{dT}$ represents the rate of susceptible individuals getting cross-infected by both infected individuals and constrained individuals per unit of time. $\frac{dI}{dT}$ represents the total number of newly infected individuals per unit of time minus the number of controlled individuals α*βI*(*t*) and σ_1_β_1_*C* represents the growth rates of infected individuals and constrained individuals per unit of time. $\frac{dC}{dT}$ represents the rate of change of constrained individuals over time, which is the difference between the number of constrained individuals and the number of individuals who no longer can transmit the virus. Here, A represents the control rate, and *AαI*(*t*) represents the rate at which constrained individuals transform into removed individuals due to recovery or receiving sufficient medical resources. $\frac{dR}{dT}$ represents the rate of change of removed individuals over time, which includes individuals who have recovered from the infection and individuals who have transformed from constrained individuals to removed individuals.

In the SICR model, the isolation rate will change according to different isolation policies. Constrained individuals, due to the impact of isolation policies, have restricted movements, and their contact rate is lower than that of regular infected individuals.

In the formula, the post-isolation contact rate σ_1_ is related to the original contact rate σ as follows: $\sigma_{1}=\frac{\sigma }{Q_{k}}$, where *Q*_*K*_ represents the regulatory intensity, and k represents different isolation policies. For example, if an open policy is adopted, meaning no strict isolation measures are implemented (i.e., *Q*_*K*_ = 1), the post-isolation contact rate σ_1_ will be equal to the original contact rate σ. In this case, it is equivalent to using the SEIR infectious disease model with an incubation period to represent the epidemic transmission process. However, when stricter isolation policies are implemented, the contact rate of constrained individuals will be reduced, thereby influencing epidemic transmission.

**Figure 3:**
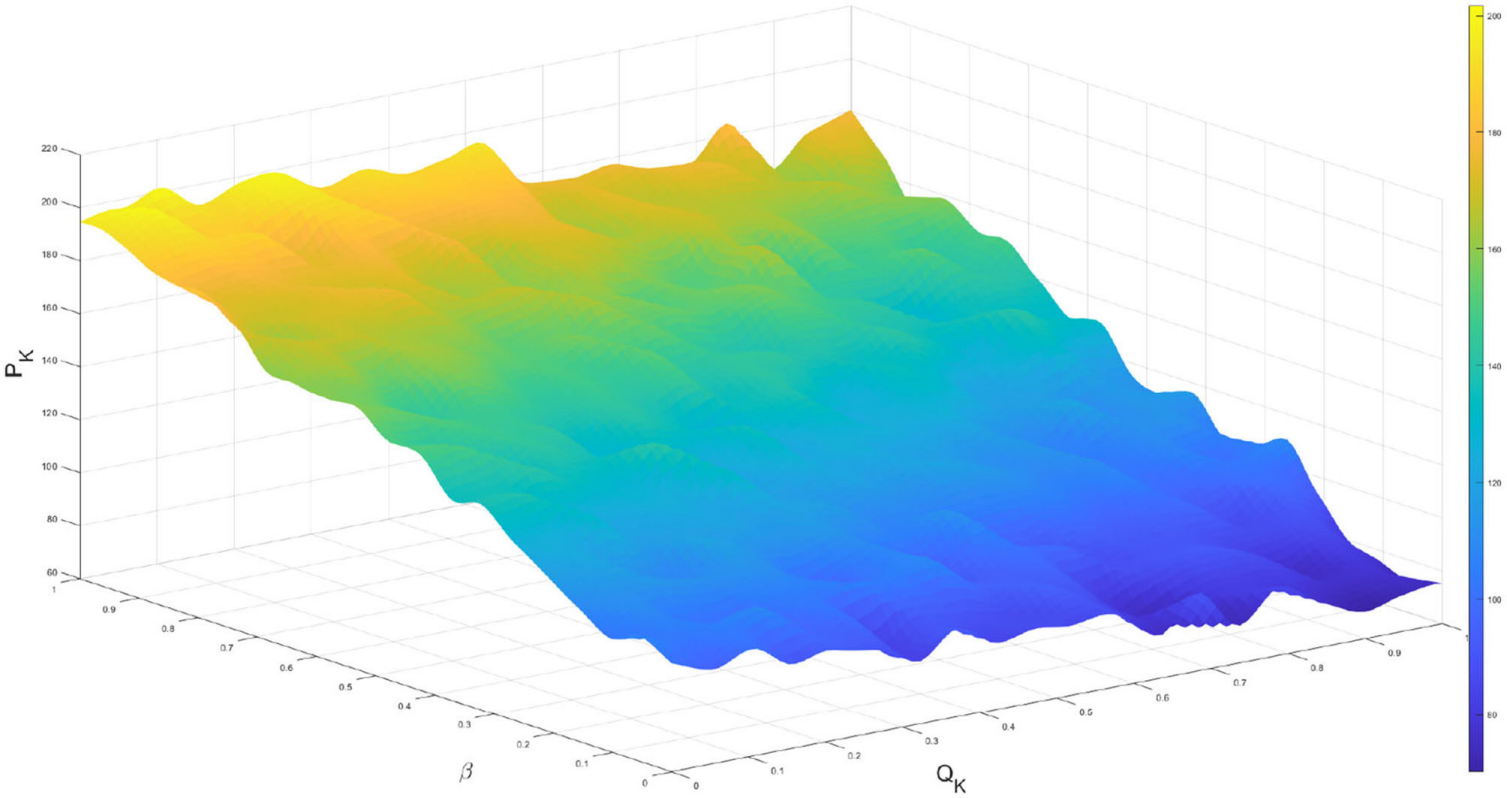
The peak number of infected individuals varies with control measures and infection rates.

To analyze the relationships among various interaction parameters, we employed statistical methods based on time-series analysis to examine the impact of *Q*_*K*_, cross infection rate β, and the peak of infected individuals (*P*_*K*_) on the dynamics of the epidemic (27). Using the improved model, we generated time-series data for the number of infected individuals under different parameter settings. As shown in Figure 3, We found that an increase in *Q*_*K*_ led to a decrease in *P*_*K*_. Additionally, we observed a positive correlation between β, indicating that increasing β would result in an increase in *P*_*K*_. Therefore, there is a negative correlation trend between regulatory intensity and infection rate.

This model set allows for a more accurate reflection of the effects of different isolation policies on the transmission process, helping to assess the effectiveness of isolation measures and devise more effective epidemic prevention strategies.

### 3.2 Swarm entropy and basic regeneration number

In epidemiology, the basic reproduction number, denoted as *R*_0_, is an essential parameter that describes the early stages of an infectious disease outbreak. It represents the expected number of secondary infections caused by introducing a single infected individual into a completely susceptible population during the individual's average infectious period. The magnitude of *R*_0_ directly influences the spread of the infectious disease within the population. In a simulation environment that does not take into account the birth and natural mortality rates, the basic regeneration number *R*_0_ can be estimated by the Equations 9, 10:


(9) $$\begin{array}{l} R_{0}=\left(1+\lambda T_{0}\right)\left(1+\lambda T_{N}\right) \end{array}$$

(10) $$\begin{array}{l} \lambda =\frac{\ln Y\left(t\right)}{t} \end{array}$$

where λ is the growth rate of infected persons, *R*_0_ is calculated by dividing the rate of infection by the rate of recovery; when *R*_0_ is <1, the number of infected persons who can be infected during the average period of infection is <1, and then the disease will gradually die out on its own and the spread of the disease will stop. When *R*_0_ is >1, the number of infected persons who can be infected during the average period of illness is >1, and the disease will continue to spread and propagate. Therefore, controlling *R*_0_ is the key to developing effective epidemic prevention measures and preventing the spread of infectious diseases. Based on the basic reproduction number, one can derive the minimum criteria that need to be met when setting various parameters in the model. For example, Young et al. (28) employed the SIQ infectious disease model to estimate the minimum coping capacity during the epidemic.

In the research, considering the incubation period of COVID-19 typically ranges from 7 to 14 days, the isolation period should be at least as long as the incubation period. Therefore, the infection period (*T*_*I*_) is set to 9 days, and the isolation period (*T*_*N*_) is set to 10 days.

In reality, it may not be feasible or practical to keep the basic reproduction number (*R*_0_) below 1 during the early stages of an epidemic, and ensuring the sustainability and flexibility of policies can be challenging. Studies have shown that when the basic reproduction number (*R*_0_) exceeds 2, the number of infections will rapidly increase, and for COVID-19, the *R*_0_ value falls within the range of (2.2, 4.2). Therefore, keeping *R*_0_ below 2 can be used as a criterion to assess the effectiveness of isolation policies and effectively control the spread of the epidemic.

To explore the impact of isolation policies on the growth rate, this study introduces the concept of structural entropy from swarm entropy theory. Swarm entropy is commonly used to analyze the information transmission efficiency among agents in a population, and the information transmission model can be represented by the structure of the SIR model. This indicates that there are similarities between the infection mechanism and information transmission mechanism, allowing the swarm entropy theory to be applied for quantifying the infection efficiency of the COVID-19 pandemic. In the context of simulating population structures, the first step is to determine the verisimilitude of the agent population ecology in the simulation environment. In Equation 11, *n* represents the sample size, *A*_*i*_ represents the observed values within a sample, which is the number of samples within a specific set, and *E*_*i*_ represents the expected values for a set, which signifies the quantity of samples in that set theoretically or under a baseline condition. A smaller *PSI* indicates a higher level of authenticity in the simulation environment.


(11) $$\begin{array}{l} PSI=\overset{n}{\underset{i=1}{\sum }}\left(A_{i}-E_{i}\right)\ln\frac{A_{i}}{E_{i}} \end{array}$$

In the same region, where the treatment rate for COVID-19 remains the same, the number of agents remains constant, and there are no significant behavioral differences, the structure of the agent population becomes the main determinant of the increase in entropy. Therefore, by analyzing the structural entropy within swarm entropy, a better understanding of the influence of different isolation policies on the information transmission efficiency among agent populations during the epidemic spread can be gained.

During the simulation process, it is possible to estimate the virus infection intensity by observing the transmission structure of the population under different isolation policies. To introduce the mechanism of swarm entropy, this study defines the swarm entropy as *H*. *H* represents the collective entropy of the agent population and serves as a measure of the diversity or disorder in the transmission structure during the epidemic spread (Equation 12):


(12) $$\begin{array}{l} H=-\underset{SIC}{\sum }P_{i}\log_{2}P_{i} \end{array}$$

In the SICR model, the swarm entropy *H* takes into account the probabilities of connections between different types of individuals in the social network. Specifically, *P*_*s*_ represents the probability of connections between susceptible individuals and other individuals, *P*_*i*_ represents the probability of connections between infected individuals and other individuals, and *P*_*c*_ represents the probability of connections between constrained individuals and other individuals. In the SICR model, we assume that the probability of connections for removed individuals is 0.

The swarm entropy *H* considers the proportions of individuals in each state by taking the logarithm of the proportions and multiplying them by their respective probabilities. A higher *H* value indicates more disorder or diversity in the individual states within the population, meaning there are more opportunities for contact between infected and susceptible individuals, resulting in higher infection intensity. Conversely, a lower *H* value indicates more uniformity or homogeneity in the individual states within the population, meaning there are fewer opportunities for contact between infected and susceptible individuals, resulting in lower infection intensity.

By introducing swarm entropy, a more comprehensive analysis of the changes in information transmission efficiency among agent populations under different isolation policies can be conducted. This enables quantification of the impact of isolation policies on the infection process, providing valuable reference for devising more effective prevention and control strategies.

We assume that the efficiency of isolation policies is directly proportional to swarm entropy, and the correlation between isolation efficiency and population structure is represented by a parameter e. The strength *Q*_*k*_ of different isolation policies is expressed as Equations 13, 14:


(13) $$\begin{array}{l} h=\frac{H}{S+I+C} \end{array}$$

(14) $$\begin{array}{l} Q_{k}=\frac{\Phi }{1+eh} \end{array}$$

The parameter e is a constant, representing the proportion of the correlation between isolation policy efficiency and population structure. *h* represents the mean structural entropy, reflecting the efficiency of information transmission within a specific population structure. Φ is a control variable to ensure that the isolation policy's efficiency remains within a normal range. The intensity of the isolation policy, represented by *Q*_*k*_, is a variable related to the swarm entropy *H*. When *Q*_*k*_ is 1, it indicates no isolation policies implemented.

By introducing parameters e and swarm entropy *H*, the intensity of isolation policies can be flexibly adjusted to better simulate the impact of different isolation policies on the epidemic transmission process in real-world scenarios. This approach can help researchers explore the effects of different isolation policies on disease transmission in simulation experiments and provide reference for formulating appropriate epidemic prevention strategies.

The model can similarly assess the strength of the isolation policies chosen for the model with knowledge of the efficiency of the isolation policies. We use the SIR model as the baseline reference model for evaluation. *Q*_0_ represents the regulatory intensity of the basic model within the simulation environment. In the SICR model, the isolation efficiency E is negatively related to the swarm entropy, p represents the unit time growth rate of the basic model. K represents the ratio of the predicted growth rate to the unit time growth rate of the basic model, as shown in Equations 15, 16:


(15) $$\begin{array}{l} R_{0}=\left(1+\frac{p}{k}T_{0}\right)\left(1+\frac{p}{k}T_{N}\right) \end{array}$$

(16) $$\begin{array}{l} K=\frac{Q_{0}}{Q_{k}}\left(Q_{0}>0\right) \end{array}$$

At the same time the effective regeneration number, D is the length of time that can be propagated, from which the isolation efficiency E can be obtained. As shown in Equation 17:


(17) $$\begin{array}{l} E=\frac{\sigma_{1}\beta D}{\left(1+\frac{p}{k}T_{0}\right)\left(1+\frac{p}{k}T_{N}\right)} \end{array}$$

### 3.3 Simulation environment

Key points from the described SICR infectious disease model and simulation mechanism are as follows.

#### 3.3.1 Epidemic transmission mechanism

In a closed environment, a limited number of individuals initially exist in a susceptible state. When some of these individuals get infected with the virus, they transition to the infectious state. Infected individuals can then become removed (recovered or deceased) through two pathways:

- Controlled state: infected individuals are subjected to isolation policies and are removed under low transmission intensity in the controlled state.
- Asymptomatic State: Special individuals (e.g., asymptomatic carriers) are not constrained by isolation policies and directly transition from the infected state to the removed state.

#### 3.3.2 Agents and experimental environment

To simulate the structure of agent groups under different isolation policies, the agents should possess a hierarchical structure. The hierarchical structure can clearly represent the social relationships between agents and facilitate the analysis of the value of group entropy. For example, under the policy of community isolation, there are two ways in which infected individuals can spread the virus:

- Infection among cohabitants: infected individuals can transmit the virus to others through contact with cohabitants.
- Infection by Higher-Level Administrators: Under the community isolation policy, there might be higher-level administrators or organizers who can also play a crucial role in spreading the virus.

The mechanisms and experimental environments described above allow the simulation and analysis of the epidemic transmission process under different isolation policies. The behavior of the agents and the setting of the environment will affect the transmission process and the value of population entropy, thus helping researchers to better understand the effect of isolation policy on the spread of infectious diseases and find the optimal epidemic prevention strategy.

**Figure 4:**
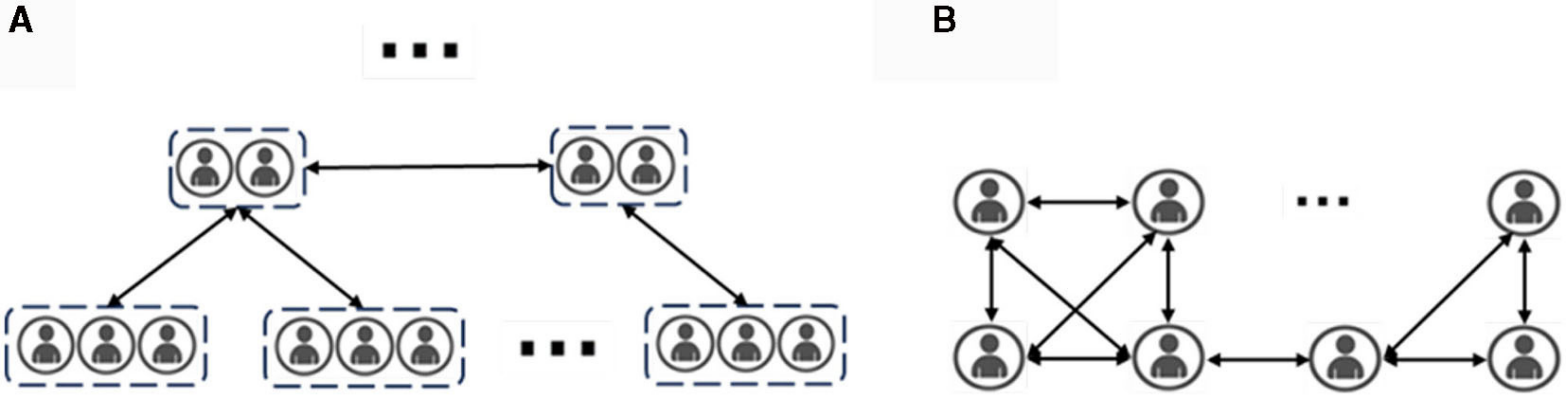
Population structure under community segregation policy. **(A)** Describes the population structure under consideration of a community segregation policy in which agents are categorized into tiers based on social relationships, with lower levels having relatively lower contact rates; **(B)** describes the structure of the population in a traditional infectious disease modeling simulation setting in which contact rates are determined only by the number of other agents in the social radius).

In the simulation of epidemic spreading under isolation policies, the behavior of agents is influenced by multiple factors, including their own state and the implemented isolation policies. As shown in Figure 4, infected individuals are subject to constraints imposed by isolation policies, which may result in the following scenarios affecting the behavior between infected and susceptible agents:

- Restricted behavior of infected individuals: infected individuals may be isolated or required to adopt specific behaviors, such as wearing masks and avoiding close contact with others. These measures aim to reduce interactions between infected and susceptible individuals, thereby lowering the risk of transmission.
- Restricted behavior of susceptible individuals: under certain isolation policies, susceptible individuals may also face restrictions, such as lockdown measures or home isolation. These measures may limit the mobility and social activities of susceptible individuals to reduce their chances of getting infected.
- Reduced interaction between infected and susceptible individuals: due to the impact of isolation policies, interactions between infected and susceptible individuals may be reduced. For instance, under social distancing policies, people may decrease their outdoor activities, resulting in fewer interactions between infected and susceptible individuals.

By incorporating the complexities of human behavior and interactions into the model, researchers can gain valuable insights into how different isolation policies affect the dynamics of the epidemic, the spread of infections, and the overall effectiveness of containment measures. Such analyses can inform evidence-based decision-making, guide the implementation of targeted interventions, and optimize the allocation of resources to control the outbreak.

## 4 Experiment

In the experiment, we used the COVID-19 pandemic as a case study to simulate the outbreak. In the experimental section, we employed the SICR model to simulate the epidemic's spread under a semi-open policy in the later stages of the outbreak and compared it with the SIR model, which does not include a controlled stage. This comparison helps us understand how the inclusion of a controlled stage affects the epidemic transmission dynamics and containment strategies.

Subsequently, we conducted sensitivity analysis experiments to assess the impact of two key factors: voluntary compliance and regulatory intensity. By varying these factors in the model, we analyzed their influence on the disease transmission process. Specifically, we observed how changes in the voluntary compliance rate and regulatory intensity affect the epidemic's spread and containment. This analysis helps identify the critical role of these factors in controlling the epidemic and provides insights into their optimal values.

The experiments in this study are conducted under the following assumptions and conditions:

- Fixed population size: the simulation assumes a fixed population of agents, and does not consider factors such as birth rates and death rates. Additionally, the model is designed to operate independently of external factors in the simulation environment.
- Reference data: the experimental data used for the simulation is based on COVID-19 transmission data from December 2022 (29). The simulation assumes a lenient isolation policy, where agents voluntarily undergo nucleic acid testing. The proportion of constrained individuals and the control rate in the model represent the willingness of people to comply with isolation measures. Besides, we adopted the OxCDGRT (Observation-based xCD-GOTCHA Real-Time) tracker as our infectious disease dataset (30). This dataset is a comprehensive tracker that contains observational data during the actual infectious disease outbreak, providing detailed information on the number of infections, recoveries and deaths. The choice of this dataset is based on its reliability in widely applied to epidemic tracking and analysis (31).
- Infection period: the COVID-19 infection period in reality ranges from 7 to 14 days, with a total infection period of around one month. In this study, we adopt a total experimental period of 25 days, and the states of infection are recorded on a daily basis.

### 4.1 Comparative experiment

We simulated the transmission of COVID-19 in a population. The simulation allows us to observe the various stages of epidemic transmission. We compared the model with the inclusion of the controlled stage with the traditional infectious disease model and set different population sizes to validate the model's reasonability.

The premise of this experiment is that people universally comply with wearing masks when going out and have sufficient medication and resources to support self-isolation. Within the specified experimental environment, we set the contact rate and the control rate, and changing these parameters can affect the results of the simulation experiment. The parameters used in this experiment are listed in Table 1. In Table 1, *V*_*p*_ represents the number of agents, with values of (200, 300, 500). *E* and *W* represent the incubation period and infection period, respectively, and follow a normal distribution within their respective ranges, representing the time it takes for agents to transition from an infected state to the controlled state C and the removed state R.

**Table 1:** Parameters of the comparative experiment.

**Parameter**	**Meaning**	**Value**
σ	Exposure rate under infection status	5
σ_1_	Contact rate under constrained state	2
β	Cross infection rate of I	0.2
β_1_	Cross infection rate of C	0.04
γ	Remove rate	0.9
*A*	Controlled ratios	0.7
α	Controlled person removal	0.2
*W*	Infectious cycle	7–12
*E*	Latency	5–10
*P*(*t*)	Total population	*V*_*p*_
*S*_0_	Initial susceptible	*V*_*p*_ − 3
*I*_0_	Initial infected	3
*C*_0_	Initial constrained	0
*R*_0_	Initial removed	0
*Q*_*k*_	Regulatory intensity	5

In the experiment, the infection rate and recovery rate were configured based on the parameters specified in (16). The epidemic cycle was modeled after the incubation period of COVID-19. The contact rate under the constrained state was set according to the number of people per household in the context of community isolation.

#### 4.1.1 SICR model simulation comparative experiment

We conducted comparative experiments on the scale of the agent population to explore the applicability of the SICR model by observing variations in the model under different agent population sizes, we also compared the number of agents in different states at the same time to analyze the pattern of pandemic development.

**Figure 5:**
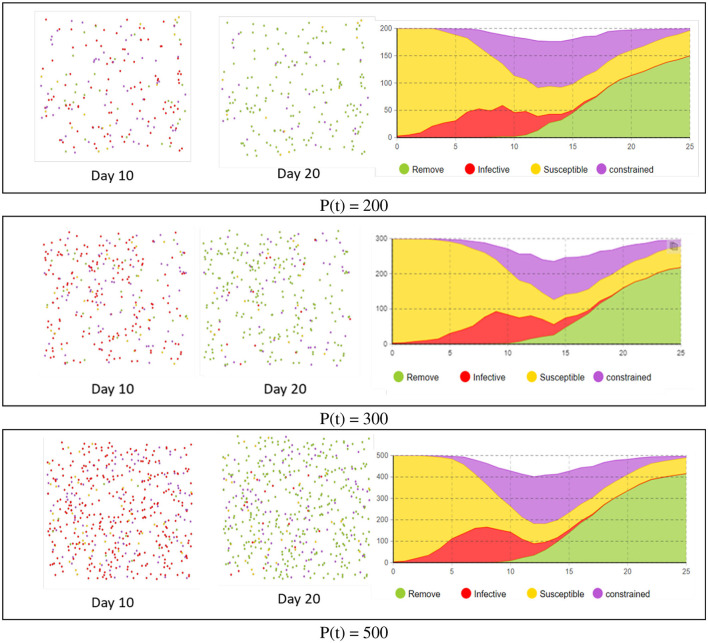
Run chart of epidemic simulation experiment.

In the epidemic transmission model, all individuals are in a closed environment, including three initially randomly distributed infected individuals. Each individual's status is represented by four different colors: susceptible individuals are in yellow, constrained individuals in purple, infected individuals in red, and recovered individuals in green. The experiment lasted for 25 time steps, with each time step corresponding to 1 day. The simulated results of the virus for the same time period are shown in Figure 5.

Throughout the entire simulation process, the size of the population (P) has influenced both the epidemic transmission period and the peak of infected individuals. When the number of individuals is small, the speed of epidemic transmission slows down, and the proportion of infected individuals decreases. Additionally, it is associated with a shorter duration for ending the epidemic. However, the overall trend remains consistent. Therefore, when comparing the experiment with actual data, the focus is on analyzing the overall trend of infections.

**Figure 6:**
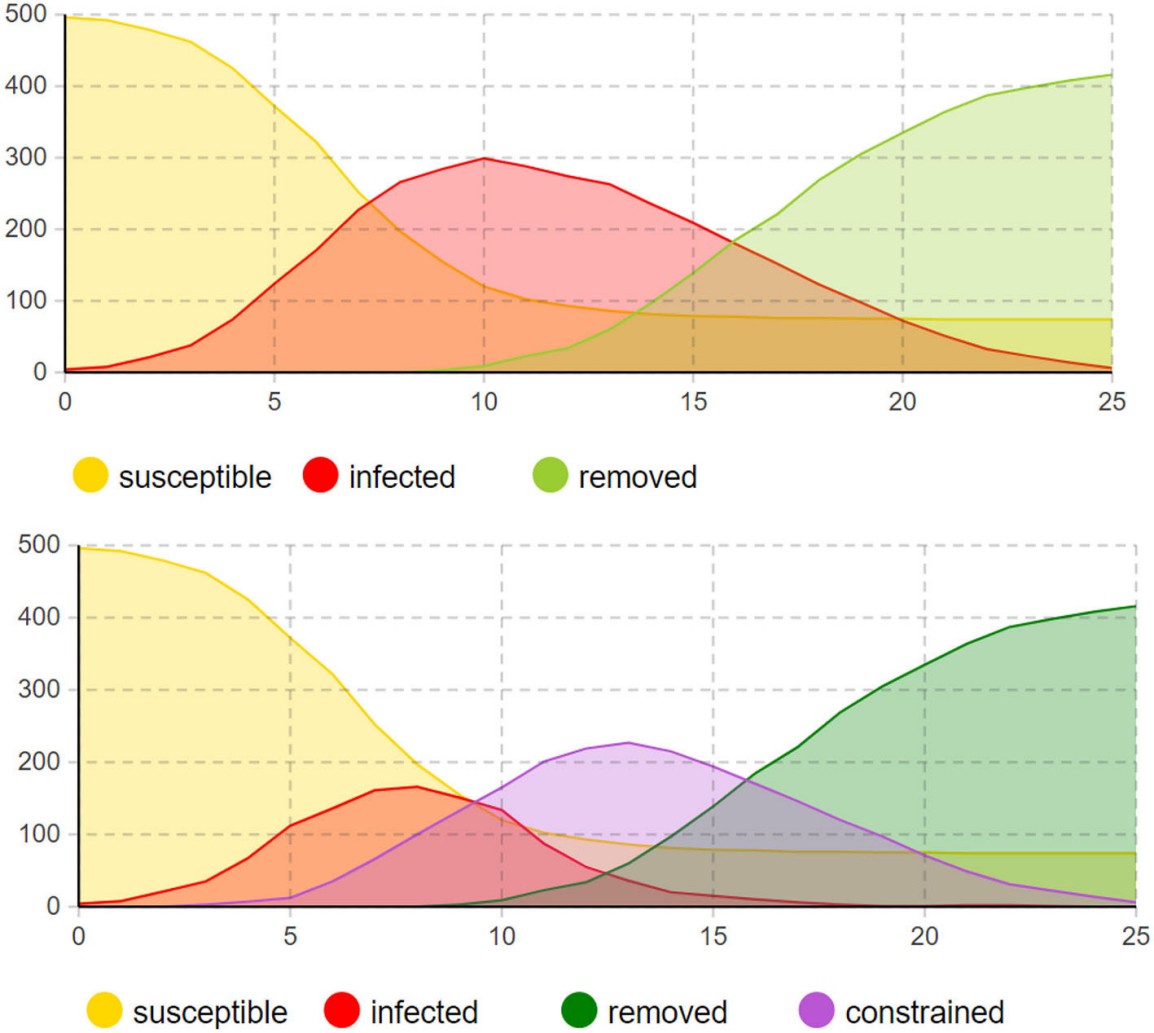
Comparison between infection status and agent status.

To clearly observe the trend of the number of infected individuals and compare it with the traditional SIR model, the experiment combined the infected and constrained individuals and calculated the total number of infected individuals when P (t) was set to (500, 300, 200). The results are shown in Figure 6. In the time axis plot for P (t) = 500, the time axis is the horizontal axis, and the total number of individuals is the vertical axis. The plot shows the number of individuals in different states, with infected individuals uniformly represented in red on the left side.

During the entire epidemic period, due to the regulation of the model by the isolation policy, the number of infected individuals reaches its peak on the seventh day, with 284 infected individuals. It then gradually decreases over the next three weeks. In the time axis plot with the introduction of the controlled stage, the constrained individuals reach their peak on the 14th day, with a growth period of about half of the total period. Compared to the changes in the number of infected individuals, the changes in the number of constrained individuals are more gradual, reflecting the inhibitory effect of the isolation policy on the growth rate of the epidemic. The experiments confirmed the adaptability of the SICR model to populations of varying sizes and intuitively reflected the trends in pandemic development.

In this experiment, we compared the SICR model with real-world data and experimental data from the traditional SIR model. We conducted an analysis of controlled ratios and regulator intensity to determine their impact on the stages of infection.

#### 4.1.2 Compared with SIR model and actual data

**Figure 7:**
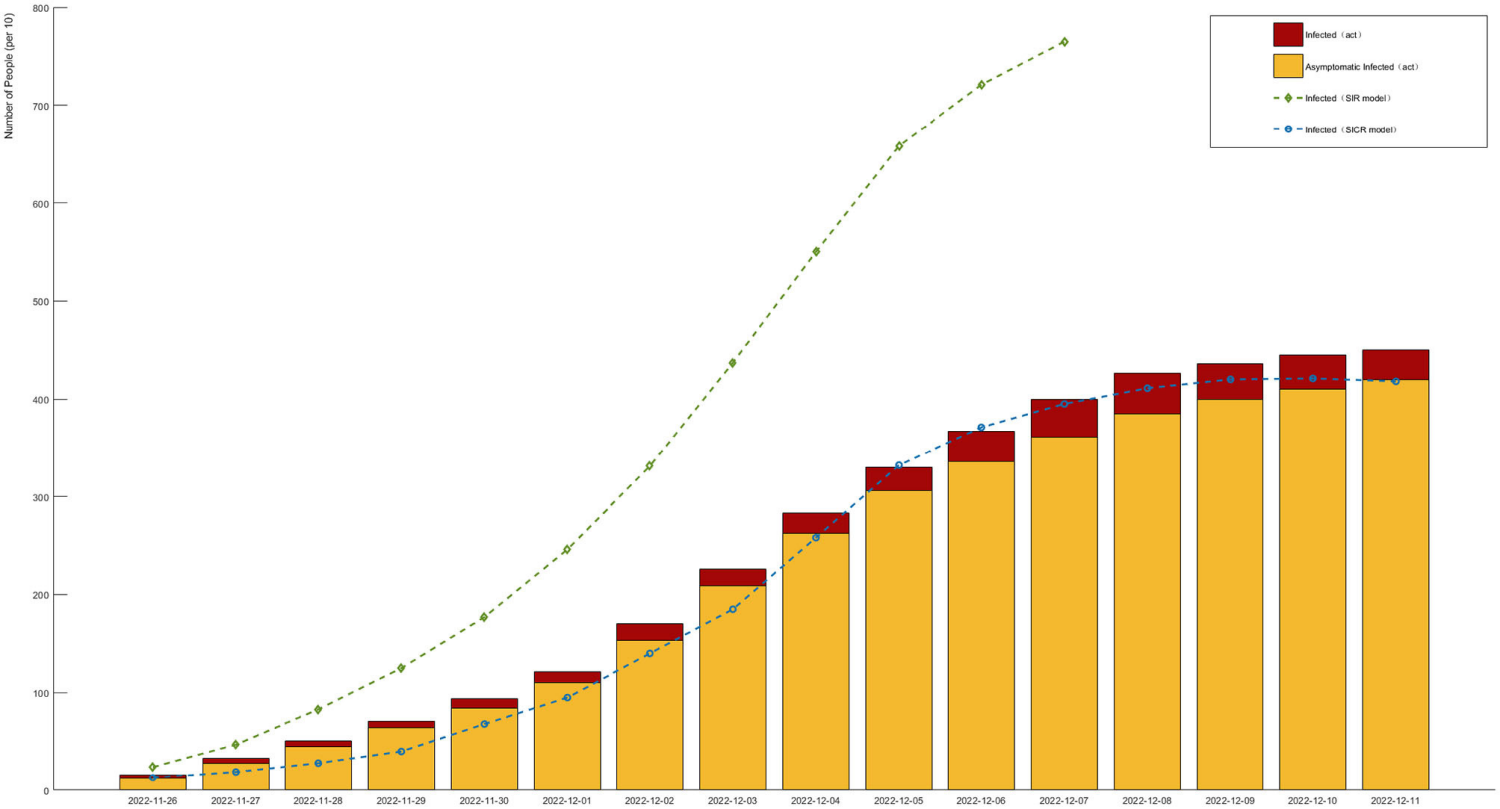
Different models compared to actual data in the infection stage (red and yellow bars represent actual data for infected and asymptomatic infections, respectively. Green and blue curves represent infection trends under the SIR and SICR models, respectively).

Based on the news released by the National Health Commission of the People's Republic of China, since December 2022, the number of positive nucleic acid tests and the positive rate showed an initial increase followed by a decrease. The number of positive cases reached its peak and then gradually declined. Under the assumption of the same infection rate, the simulation was conducted by adopting a non-regulatory attitude toward asymptomatic infections. The fit between the SICR model, traditional infectious disease model, and actual data is shown in Figure 7.

According to the experimental results, the introduction of the controlled stage in the SICR model shows a consistent overall trend with the survey data. However, when using the traditional SIR model, even considering the impact of isolation policies on the infection rate, it still leads to errors in predicting the proportion of infected individuals. The SIR model also tends to produce larger fluctuations and overestimates the peak of infections, almost reaching the total number of samples. The SICR model exhibits clear advantages in predicting the dynamic trend of infectious diseases. Although the overall infection cycle in the simulation is shorter than in reality due to limitations in the simulation environment and the number of agents, the model can still better capture the fluctuation patterns and turning points of the infectious disease. For example, the SICR model can accurately predict the outbreak period and the proportion of infections under isolation policies for COVID-19, whereas the SIR model fails to accurately reflect these changes.

**Figure 8:**
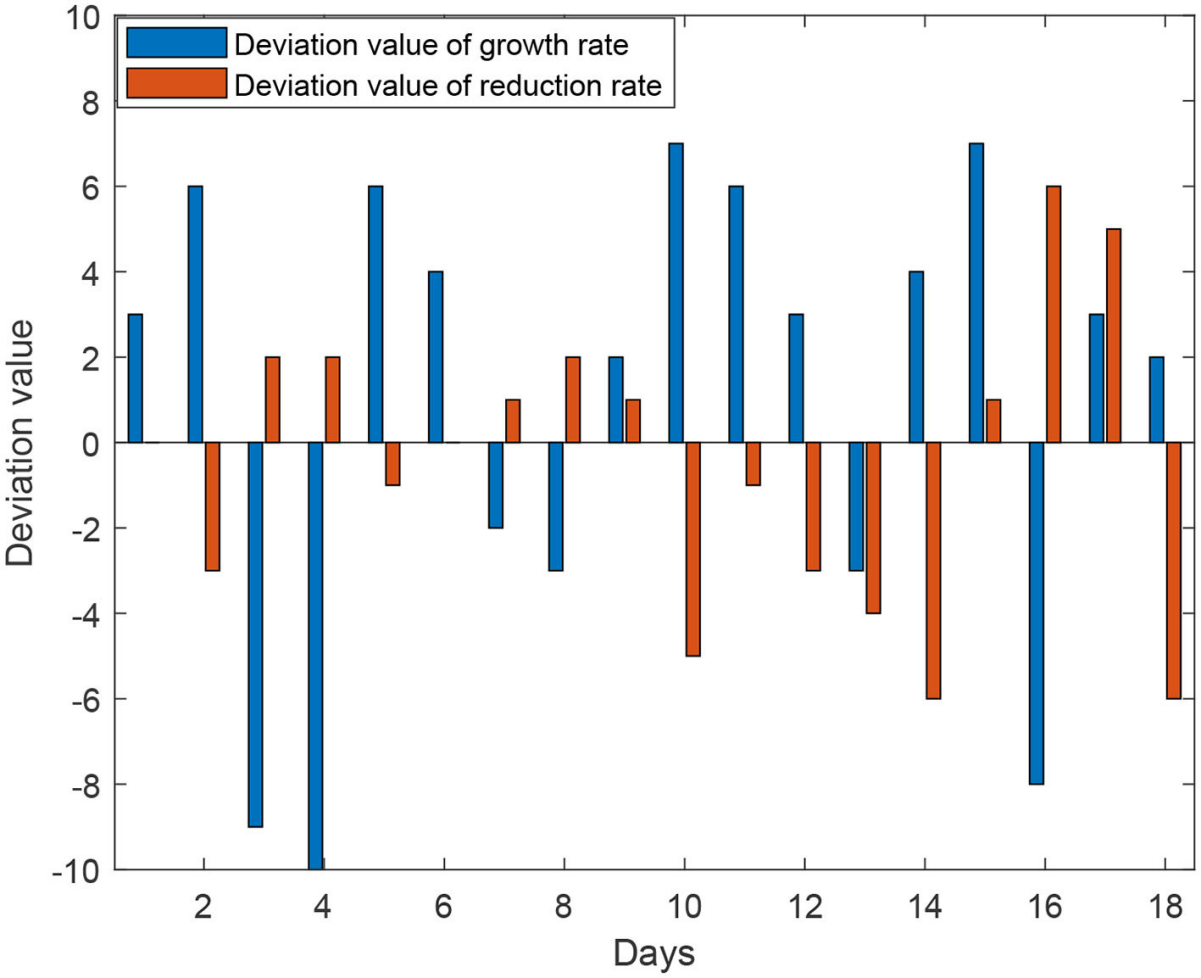
Deviation value between simulation results of SICR model and actual data.

The experiment also included the calculation of the deviation between the predicted growth rate and the recovery rate with respect to the actual data, as shown in Figure 8. Figure 8 presents a bar chart with positive and negative bars, describing the deviation between the predicted infection growth rate and the actual data. The vertical axis represents the deviation percentage, ranging from −10 to 10%, while the horizontal axis represents the days. The deviation in the infection rate is controlled within 6%, ensuring the accuracy of the model's predictions. On the other hand, the recovery rate shows larger fluctuations, but this does not affect the accuracy of the simulation results.

The results demonstrate that the SICR model accurately captures the infection growth rate, as the deviation remains within an acceptable range, validating the reliability and robustness of the SICR model in capturing the dynamics of infectious diseases. Accurate prediction of the infection growth rate provides valuable insights for decision-making and intervention strategies in disease control and prevention. We also utilized swarm entropy to evaluate the simulated isolation policies. We compared the growth rates of the SIR model and the SICR model, according to formula (17), rounded the isolation efficiency value to 6. This can serve as a benchmark for specifying other isolation policies.

To verify the credibility of our proposed multi-agent modeling method in predicting and evaluating the effects of isolation policies on infectious disease transmission, we conducted a series of experiments, comparing the actual infectious disease data and our model simulation results of the United States, Germany, Japan, and China. We chose these four countries because they have significant differences in population structure, health system and isolation policies, which provide a more comprehensive validation.

We collected the actual data of each country at the beginning stage of the epidemic transmission. We set the model input parameters based on these actual data. Then, we conducted multiple simulation experiments, simulating the transmission situation of each country under different isolation policies. To ensure the accuracy of the model, we adjusted the key parameters in the model according to the actual situation, such as infection rate, recovery rate. Meanwhile, we evaluated the setting of pandemic efficiency-related parameters by referencing the Stringency Index compiled in the dataset. The Stringency Index is a composite measure derived from nine response indicators, including school closures, workplace closures, and travel bans.

**Figure 9:**
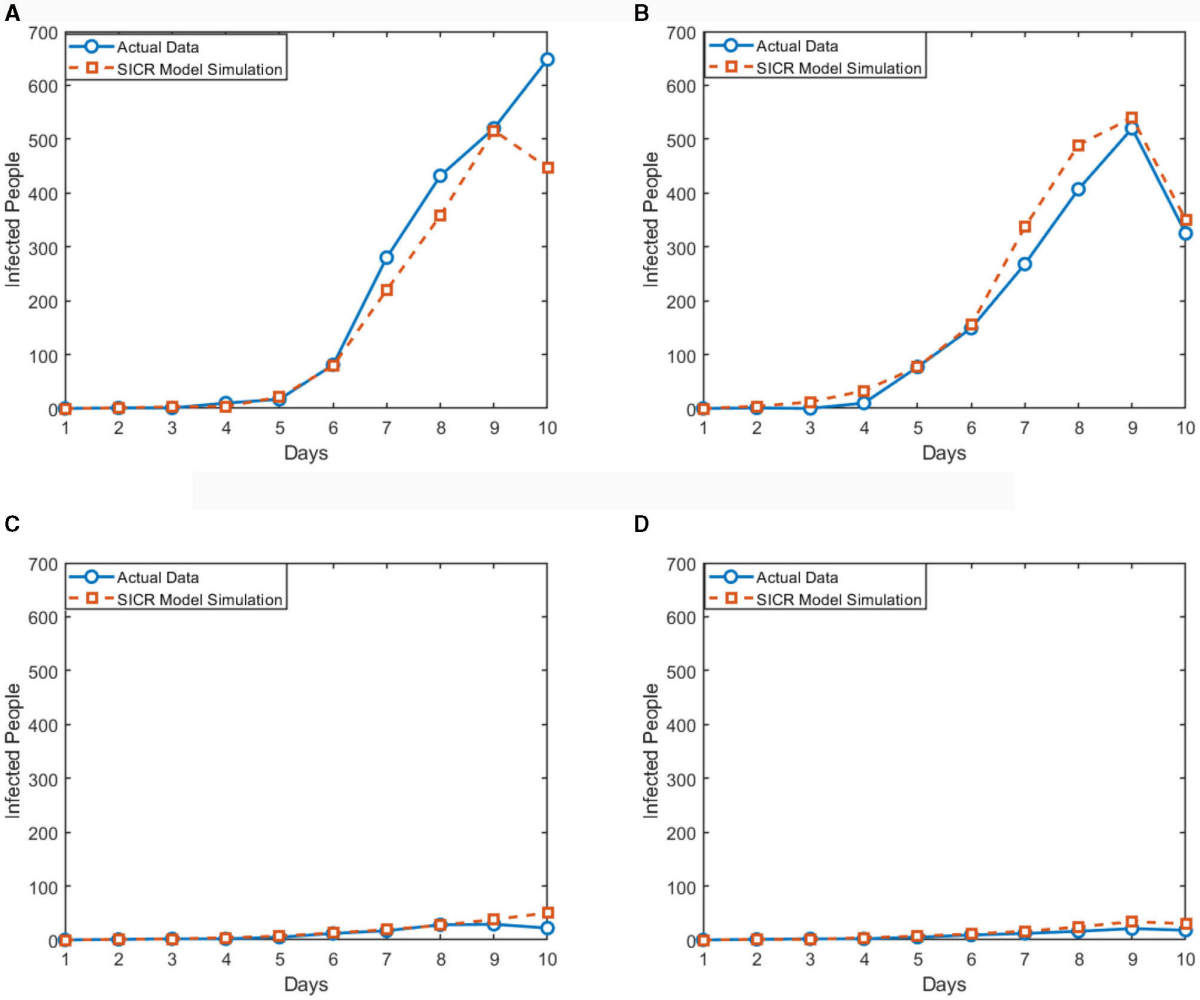
Comparison of SICR model simulation and actual data in different countries. **(A)** The United States. **(B)** Germany. **(C)** Japan. **(D)** China.

As shown in Figure 9, during the initial phase of the pandemic, the impact of isolation policies varied significantly among countries due to policy differences. We selected four countries, each adopting either strict or relatively lenient policies, to illustrate the actual number of new infections and compared it with the curves simulated by the SICR model. By contrasting the observed trends with model predictions, we could assess the adaptability and accuracy of the model in predicting disease spread in different countries. Specifically, Japan and China, implementing strict isolation policies, exhibited a notably prolonged infectious period, providing more favorable conditions for case management. Meanwhile, influenced by *Q*_*K*_, Germany experienced an earlier recovery of individuals, thereby shortening the period of rising new infections. Despite the United States adopting similar epidemic prevention policies, resource shortages and issues such as protests led to a delay in the impact of *Q*_*K*_, resulting in disparities with the model results. In our subsequent work, our goal is to introduce an algorithm for real-time adjustment of the infection rate to enhance the adaptability of the model.

### 4.2 Sensitivity analysis experiment

Sensitivity analysis is a powerful tool for quantifying the impact of parameter changes on the behavior of a system (23). In the SICR model, two important parameters affect the process of infectious diseases. The control strength *Q*_*k*_ represents the external environment's impact on the model, which influences the infection rate and control rate to affect the infectious disease model. The controlled ratio A represents the impact of the individual's willingness on the model, which changes the epidemic transmission trend by affecting the proportion of constrained individuals. Therefore, in this experiment, sensitivity analysis was conducted separately for the control strength *Q*_*k*_ and the controlled ratio A, using the number of infected individuals as the uncertainty for the analysis.

#### 4.2.1 Sensitivity analysis of controlled ratio

**Figure 10:**
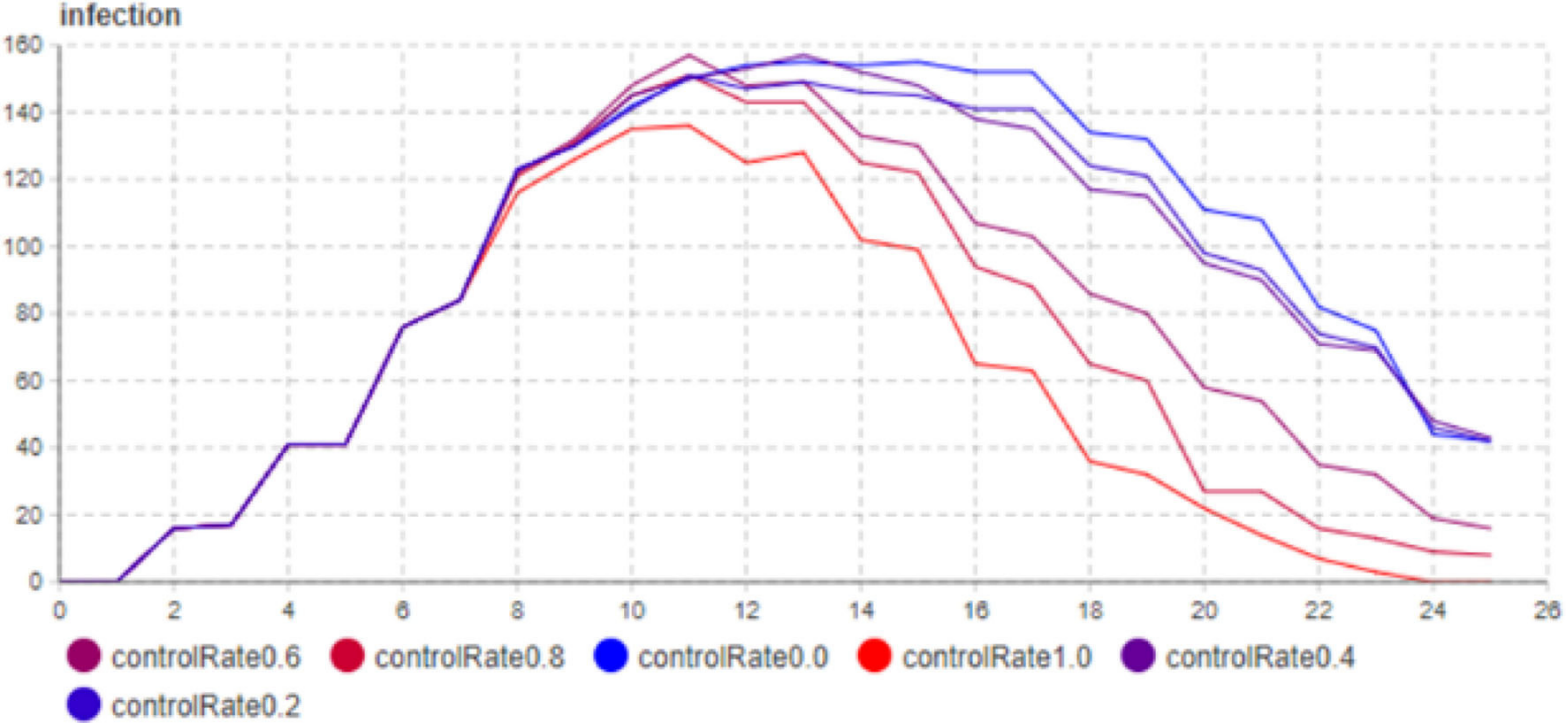
Sensitivity analysis experiment of constrained rate.

The experiment used time T as the x-axis and the number of individuals in the infected state as the y-axis. The simulation results generated data were input into the sensitivity model to plot the curves and data for the number of individuals in the infected state. The controlled ratio A ranged from Max = 1 to Min = 0, with a step size of 0.2. In order to conduct the sensitivity analysis shown in Figure 10, a total of six experiments were performed, and the corresponding data for each experiment was displayed in the curves. The parameter settings are as indicated in Table 2.

**Table 2:** Parameters of the sensitivity analysis experiment.

**Parameter**	**Meaning**	**Value**
*P*(*t*)	Total population	500
*S*_0_	Initial susceptible	497
*I*_0_	Initial infected	3
*C*_0_	Initial constrained	0
*R*_0_	Initial removed	0
*A*	Constrained rate	0–1
*Q*_*k*_	Regulatory intensity	1–5

Considering the closed social environment, population size, population mobility, and high infectivity of the virus, the sample size for sensitivity analysis was set to 500 individuals. The number of infections on the 10th day and the 16th day were separately recorded. As shown in Table 3, the controlled ratio does not significantly affect the trend of epidemic transmission in the first 8 days. However, after 8 days, as the controlled ratio increases, the number of infected individuals reaching the peak decreases, and this change is more significant in the range of A = (0, 0.4).

**Table 3:** Results of the of controlled ratio sensitivity analysis experiment.

**Experiment**	**Constrained rate**	**10 days**	**16 days**
Run0	0	140	115
Run1	0.2	143	100
Run2	0.4	141	97
Run3	0.6	140	60
Run4	0.8	140	28
Run5	1.0	137	20

Based on the results of the sensitivity analysis on the controlled ratio, the following conclusions can be drawn:

- The impact of the controlled ratio on the transmission process is mainly evident during the decline phase of the number of infected individuals, and it can also have a certain effect on the peak value of the infection.
- The residents' willingness to comply with the control measures is directly proportional to the value of the controlled ratio. The stronger the compliance of the individuals, the better the epidemic can be controlled. In this experiment, when the controlled ratio of the individuals reaches 1, the decline period of the epidemic is shortened by half. Therefore, when implementing policies, it is essential to take into account the residents' willingness to cooperate and comply with the measures.

#### 4.2.2 Sensitivity analysis of regulatory intensity

**Figure 11:**
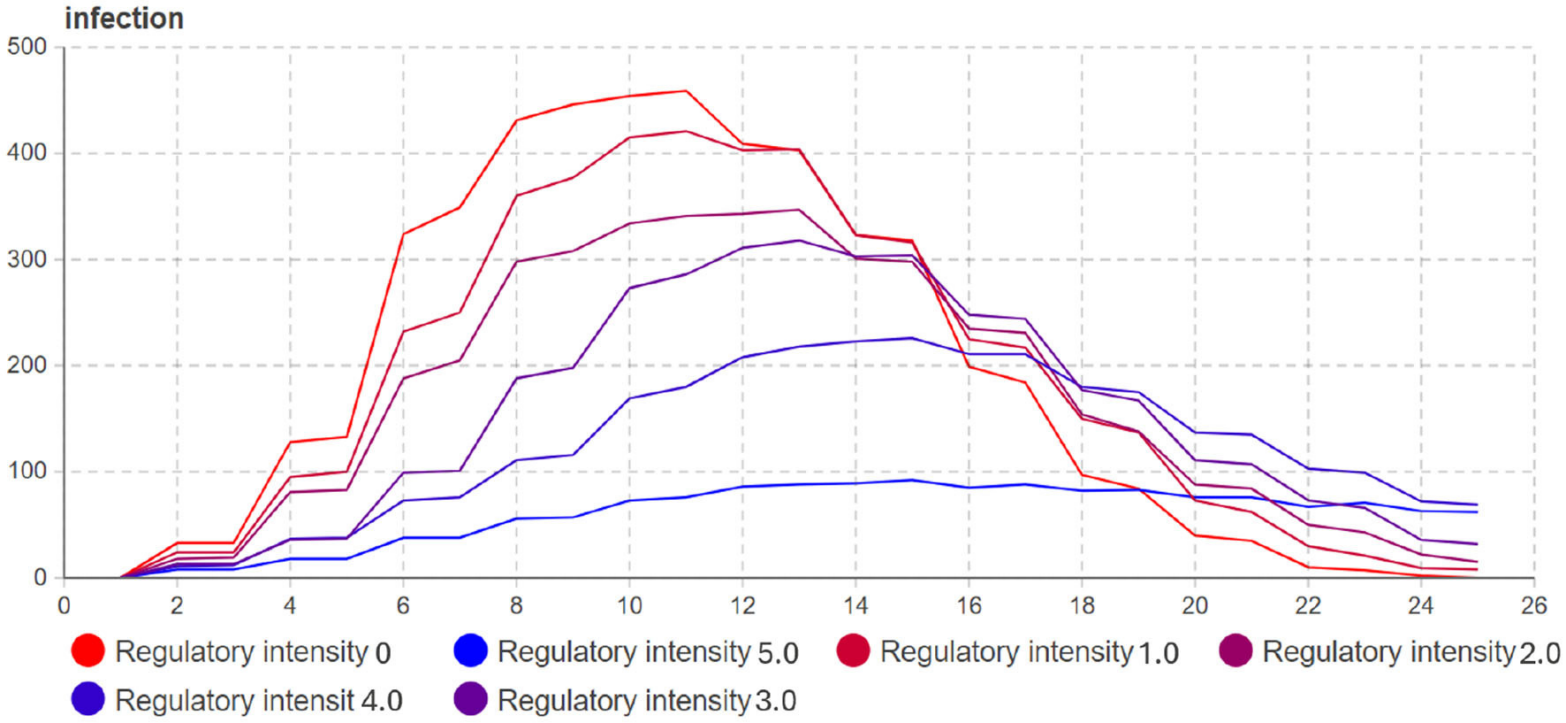
Sensitivity analysis experiment of regulatory intensity.

Based on the sensitivity analysis results on the controlled infection rate (*Q*_*k*_), the following observations can be made: As shown in Figure 11 and Table 4, the impact of the controlled infection rate on the transmission process is mainly evident in the later stages of the infection cycle. It has a smaller effect on the initial phases of the epidemic; As the controlled infection rate (*Q*_*k*_) increases, the growth rate of infected individuals slows down, and the duration of the infection cycle becomes longer; When there is a significant difference in infection intensity (i.e., high *Q*_*k*_ values), the number of infected individuals at the peak decreases.

**Table 4:** Results of the regulatory intensity sensitivity analysis experiment.

**Experiment**	**Regulatory intensity**	**10 days**	**16 days**
Run0	1	433	320
Run1	1.2	360	300
Run2	1.4	300	300
Run3	1.6	290	290
Run4	2.1	112	225
Run5	3	50	95

Based on the results from both the sensitivity analysis experiments on the controlled infection rate (A) and the regulatory intensity (*Q*_*k*_), the following conclusions can be drawn:

- The impact of the controlled infection rate (A) on the transmission process is mainly evident in the phase of increasing infected individuals, and it also affects the peak number of infections. Larger values of A result in more significant effects on the transmission dynamics.
- Increasing the regulatory intensity (*Q*_*k*_) is a direct means to control the growth trend of the epidemic. However, in reality, high regulatory intensity may lead to reduced willingness of individuals to comply with the measures. Therefore, the regulatory intensity should be set at a minimum level that still significantly affects the growth of the infected population.

Overall, the combined results suggest that both the controlled infection rate (A) and the regulatory intensity (*Q*_*k*_) play crucial roles in controlling the transmission of the epidemic. A balance needs to be struck between effective control and public acceptance of the measures. Finding the optimal values for A and *Q*_*k*_ can help design efficient and feasible epidemic control strategies.

## 5 Conclusion

The study presents an improved SIR model to simulate the impact of isolation policies on epidemic transmission. The results demonstrate that utilizing population entropy allows for a quantitative analysis of the effectiveness of various isolation policies, enabling the integration of different policies into the infectious disease model to adapt to diverse scenarios. Furthermore, through a comprehensive analysis of regulatory intensity and controlled infection rate, we emphasize the importance of considering residents' willingness in specifying isolation policies. The intensity of the policies can influence the voluntary compliance of the population, and inappropriate policies may lead to a significant extension of the epidemic transmission cycle. The primary contributions of this paper are as follows:

- We introduce the concepts of regulatory intensity and controlled infection rate into the improved SIR model, accounting for the interaction between individuals and the external environment, as well as the influence of individuals' own willingness on the infectious process. By incorporating these two essential parameters, the model can effectively regulate isolation policies and residents' compliance without requiring complex model structures to accommodate different policies.
- The application of the population entropy mechanism ensures the credibility of the simulated experimental data, providing a valid means to verify the efficiency of epidemic transmission. Moreover, the estimation of regulatory intensity through structural entropy validates the applicability of this approach to simulate the development trends of epidemics. Given the similarities between epidemic models and information transmission models, this methodology can also be extended to simulations in other contexts, such as network information dissemination.

The study has made significant contributions by incorporating isolation policies into the improved SIR model and quantitatively analyzing their impact using population entropy. The model considers both external control factors and individual compliance, offering a flexible framework for different isolation strategies. However, limitations include the assumption of a closed environment and homogeneous population, which may not fully reflect real-world complexities. Future research should consider demographic dynamics, individual heterogeneity, and real-time data updates to enhance the model's accuracy. Additionally, expanding the scope to regional or global interactions and integrating vaccination and variant effects will improve the model's applicability.

## Data availability statement

The original contributions presented in the study are included in the article/supplementary material, further inquiries can be directed to the corresponding author.

## Author contributions

JX: Conceptualization, Data curation, Methodology, Supervision, Validation, Writing – review & editing. YG: Conceptualization, Data curation, Formal analysis, Methodology, Project administration, Validation, Visualization, Writing – original draft. MZ: Supervision, Validation, Writing – review & editing.
